# Habitual Spectacle-Corrected Distance Visual Acuity and Axial Elongation in School-Based Myopia Screening: A Retrospective Cohort Study

**DOI:** 10.3390/healthcare14142076

**Published:** 2026-07-10

**Authors:** You-Ruo Zhang, Qiu-Lin Mi, Huan Xiao, Ying-Ying Nie, Yi-Chun Chai, Ting Li, Jun-Guo Duan

**Affiliations:** 1 School of Ophthalmology, Chengdu University of Traditional Chinese Medicine, Chengdu 610075, China; zhangyouruo@cdzyydx1.wecom.work (Y.-R.Z.); opthal66@cdutcm.edu.cn (Y.-Y.N.); chaiyc2001@stu.cdutcm.edu.cn (Y.-C.C.); 2 Sichuan Provincial Key Laboratory of Traditional Chinese Medicine Eye Health, Chengdu 610075, China; 3 Independent Researcher, Chengdu 610000, China; leety0725@163.com

**Keywords:** school-based myopia screening, axial elongation, habitual spectacle-corrected distance visual acuity, functional under-correction, single-vision spectacles, propensity score matching

## Abstract

**Background/Objectives**: Progressive axial elongation is a key structural indicator of myopia progression. In school-based screening, repeated ocular biometry may not always be feasible; routinely collected visual-acuity information may support follow-up prioritization. This study examined the association between stable habitual spectacle-corrected distance visual-acuity profiles and annualized axial length growth. **Methods**: This retrospective real-world cohort study used the Chengdu school-based myopia screening database. Children aged 7.0–9.9 years with screening—defined as low-to-moderate myopia and three consecutive screening visits—were classified as adequately corrected (ACG), functionally under-corrected (UCG), or uncorrected myopia (UMG) according to stable spectacle-wearing and visual-acuity profiles. The primary outcome was annualized axial length growth from baseline to the second follow-up (ΔAL). The primary analysis used 1:1 propensity-score matching; robustness was assessed using interval-specific outcomes, alternative caliper widths, and full-cohort multivariable regression. **Results**: The final cohort included 4152 children: 391 ACG, 551 UCG, and 3210 UMG. Matching retained 313 ACG–UCG pairs and 387 UCG–UMG pairs. ΔAL was lower in the ACG than in the UCG groups (0.224 vs. 0.362 mm/year; mean difference, −0.139; 95% CI, −0.165 to −0.112; *p* < 0.001). No statistically significant ΔAL difference was detected between the UCG and UMG (*p* = 0.412). Sensitivity analyses yielded consistent findings. **Conclusions**: Stable habitual spectacle-corrected distance visual-acuity profiles were associated with annualized axial length growth. Achieved visual acuity under habitual spectacle-wearing conditions may provide information beyond spectacle-wearing status alone; however, these observational findings do not establish equivalence, predictive utility, or causality.

## 1. Introduction

Childhood myopia has become an important public health and clinical concern because of its high prevalence, early onset, and potential contribution to long-term ocular complications [1,2,3]. Progressive axial elongation is the principal structural manifestation of myopia progression and is closely related to the risk of future sight-threatening ocular morbidity. Therefore, identifying school-aged children who may require closer follow-up is important for eye health management and follow-up planning.

School-based myopia screening provides an opportunity to identify children with myopia who may require further ophthalmic monitoring. However, repeated ocular biometry for all children at every screening point may not be feasible because of limitations in equipment availability, cost, personnel, and implementation feasibility. In many real-world school-based myopia screening programs, visual-acuity testing remains the most widely available and scalable assessment [4]. Clarifying how routinely collected visual acuity relates to axial elongation may therefore improve follow-up prioritization in school-based myopia screening.

In school health records and screening reports, spectacle wearing is often recorded as a simple binary indicator. However, wearing spectacles does not necessarily indicate effective functional correction [5]. A previous large-scale school-based screening study in Chengdu also showed that, despite relatively high spectacle-wearing rates among children and adolescents, the effective correction rate based on standard visual acuity was only 50.5% [1]. Children who wear spectacles but have reduced habitual spectacle-corrected distance visual acuity may not be distinguished by a binary record of spectacle wear.

Therefore, this study examined the association between stable habitual spectacle-corrected distance visual-acuity profiles across three consecutive screening visits and annualized axial length growth. We compared children with adequate correction, functional under-correction, and uncorrected myopia using propensity score matching, with full-cohort multivariable regression as a sensitivity analysis.

## 2. Materials and Methods

### 2.1. Study Design and Ethics

This retrospective real-world cohort study used routinely collected data from a large school-based myopia screening program in Chengdu, Sichuan Province, China. Longitudinal data were extracted for children aged 7.0–9.9 years who participated in three consecutive screening visits between 19 November 2019 and 18 August 2022. The study was reported in accordance with the Strengthening the Reporting of Observational Studies in Epidemiology (STROBE) guidelines for cohort studies. The analysis focused on stable correction-status and visual-acuity profiles observed across three consecutive visits and their associations with annualized axial length growth.

The study was conducted in accordance with the Declaration of Helsinki and was approved by the Ethics Committee of the Affiliated Eye Hospital of Chengdu University of Traditional Chinese Medicine (approval No. 2019YH-007; approval date: 19 September 2019). Because this was a retrospective analysis of an existing school-based screening database and involved no additional intervention, the requirement for written informed consent was waived by the ethics committee.

### 2.2. Eligibility Criteria

The initial inclusion criteria were as follows: (1) age 7.0–9.9 years at baseline; (2) right-eye non-cycloplegic spherical equivalent refraction (SER) > −6.00 D and ≤−0.50 D at baseline; and (3) availability of records from three consecutive school-based myopia screening visits. Participants were excluded if they had a history of ocular surgery, ocular trauma, or organic ocular disease; incomplete or nonconsecutive records; extreme biologically implausible values; or recorded use of myopia-control interventions other than single-vision spectacles during follow-up, including atropine, contact lenses, orthokeratology, or myopia-control spectacle lenses.

After the initial eligibility criteria were applied, longitudinal consistency screening was conducted. Only children who fulfilled the criteria for the same correction-status and visual-acuity group at baseline, the first follow-up, and the second follow-up were retained in the final analytic cohort, as described in Section 2.3.

### 2.3. Longitudinal Consistency and Group Definitions

Only right-eye measurements were used for group classification and subsequent statistical analyses to avoid inter-eye correlation. Therefore, SER, corrected distance visual acuity (CDVA), and uncorrected distance visual acuity (UCVA) used for group classification were obtained from right-eye screening measurements.

Participants were retained in the final analytic cohort only if they fulfilled the criteria for the same correction-status and visual-acuity group at baseline (T0), the first follow-up (T1), and the second follow-up (T2). This longitudinal consistency requirement was applied to reduce misclassification arising from transient changes in spectacle use, visual acuity, or measurement variability. Consequently, the groups represented stable longitudinal correction and visual-acuity profiles rather than exposure groups defined exclusively at baseline.

Participants were classified into three mutually exclusive groups. The adequately corrected group (ACG) included children who wore single-vision spectacles (SVS) at all three visits, met the screening-defined low-to-moderate myopia criterion, and achieved normal habitual spectacle-corrected distance visual acuity at all three visits, defined as CDVA ≤ 0.0 logMAR. Children who wore SVS at all three visits and had habitual spectacle-corrected distance visual acuity > 0.0 logMAR at each visit were operationally classified as the functionally under-corrected group (UCG). In this study, “functionally under-corrected” refers only to reduced habitual distance visual acuity with existing spectacles, rather than confirmed refractive under-correction or its underlying etiologies. The uncorrected myopia group (UMG) included children who did not wear spectacles at any of the three visits, met the same myopia criterion, and had UCVA > 0.0 logMAR at all three visits.

**Figure 1:**
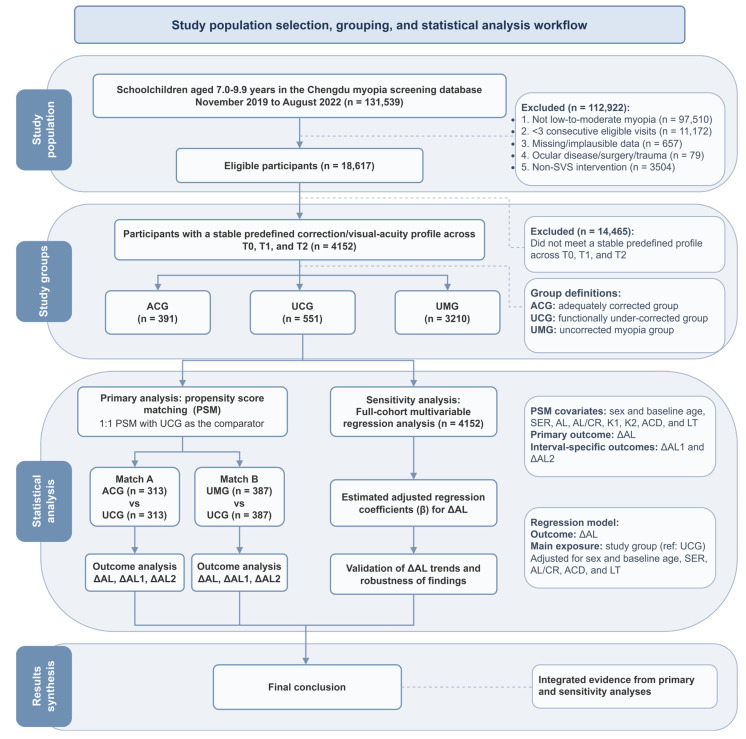
Study population selection, longitudinal group classification, and statistical analysis workflow. SVS, single-vision spectacles; ACG, adequately corrected group; UCG, functionally under-corrected group; UMG, uncorrected myopia group; PSM, propensity score matching; SER, spherical equivalent refraction; AL, axial length; AL/CR, axial length-to-corneal radius ratio; K1/K2, flat and steep keratometry; ACD, anterior chamber depth; LT, lens thickness; T0, baseline; T1, first follow-up; T2, second follow-up; ΔAL1, annualized axial length growth from baseline to the first follow-up; ΔAL2, annualized axial length growth from the first follow-up to the second follow-up; ΔAL, overall annualized axial length growth from baseline to the second follow-up.

Because information on spectacle prescription, lens age and condition, optical centration, and objective daily wearing time was unavailable, classification in the UCG did not establish the specific cause of functional under-correction. The participant selection, longitudinal grouping, and statistical analysis workflow is shown in Figure 1.

### 2.4. Ophthalmic Examinations

All examinations were performed on school premises by trained optometrists or screening personnel following standardized quality-control procedures. UCVA was measured at 5 m using a standardized logarithmic visual-acuity chart (GB/T 11533-2011) [6] under illumination greater than 500 lux. For spectacle wearers, habitual spectacle-corrected distance visual acuity was measured while children wore their own daily spectacles, and spectacle-wearing status and lens type were recorded at each visit by direct inspection and student confirmation. Visual acuity values were expressed in logMAR units for analysis. Anterior segment evaluation was performed using slit-lamp biomicroscopy.

Non-cycloplegic autorefraction was performed using a Topcon KR-1 (Topcon Corporation, Tokyo, Japan) or a Nidek ARK-1 (Nidek Co., Ltd., Gamagori, Japan). Three measurements were obtained for each eye; if any two readings differed by more than 0.50 D, additional triplicate measurements were obtained until the variation among repeated measurements was less than 0.50 D. SER was calculated as sphere plus one-half cylinder.

Axial length (AL), corneal curvature (K1 and K2), anterior chamber depth (ACD), and lens thickness (LT) were measured using the Sovision SW-9000 optical biometer (Sovision Medical Technology Co., Ltd., Chongqing, China). All devices were calibrated according to the manufacturers’ recommendations, and measurements were repeated when quality-control criteria were not met.

### 2.5. Outcome Definitions

Annualized axial length growth was used as the outcome measure of myopia progression to account for differences in actual follow-up intervals. T0, T1, and T2 represented baseline, the first follow-up, and the second follow-up, respectively. The primary outcome was ΔAL, defined as overall annualized axial length growth from baseline to the second follow-up. Two interval-specific outcomes were further calculated: ΔAL1, defined as annualized axial length growth from baseline to the first follow-up, and ΔAL2, defined as annualized axial length growth from the first follow-up to the second follow-up.

ΔAL1 = (AL_T1 − AL_T0)/[(Date_T1 − Date_T0)/365.25].

ΔAL2 = (AL_T2 − AL_T1)/[(Date_T2 − Date_T1)/365.25].

ΔAL = (AL_T2 − AL_T0)/[(Date_T2 − Date_T0)/365.25].

### 2.6. Statistical Analysis

All data management and statistical analyses were performed using Python 3.9.6. Continuous variables are presented as means ± standard deviations and categorical variables as counts and percentages. Complete-case analysis was used, and all tests were two-sided, with *p* < 0.05 considered statistically significant. Baseline characteristics of included and excluded children after longitudinal-consistency screening were compared using Welch’s *t* tests or Pearson’s chi-square test, with absolute standardized mean differences used to assess between-group differences.

Propensity scores were estimated separately for Match A (ACG vs. UCG) and Match B (UCG vs. UMG) using L2-penalized logistic regression implemented with the LogisticRegression function in scikit-learn. The inverse regularization strength parameter was set to C = 1.0, the lbfgs solver was used, and no data-driven tuning or cross-validation of the penalty parameter was performed. Baseline sex, age, SER, AL, AL/CR, K1, K2, ACD, and LT were included, whereas variables defining correction status and visual acuity were excluded. Greedy 1:1 nearest-neighbor matching without replacement was performed with exact matching on sex and a caliper width of 0.20 standard deviations of the logit of the propensity score. Propensity-score overlap and covariate balance were assessed before and after matching; an absolute standardized mean difference < 0.10 indicated adequate balance. Matched outcomes were compared using paired-sample *t* tests.

Sensitivity analyses included interval-specific outcomes (ΔAL1 and ΔAL2), alternative caliper widths of 0.10, 0.15, and 0.25, and full-cohort multivariable linear regression. The reduced regression model included study group, sex, age, SER, AL/CR, ACD, and LT. For the full-cohort regression, 1000 bootstrap resamples were drawn at the participant level with replacement; the reduced model was refitted in each resample, and 95% confidence intervals were calculated using the percentile method, defined by the 2.5th and 97.5th percentiles of the bootstrap distribution.

## 3. Results

### 3.1. Study Population, Follow-Up Intervals, and Baseline Characteristics

A total of 131,539 children aged 7.0–9.9 years were identified from the Chengdu school-based myopia screening database. After the initial eligibility screening, 18,617 children remained. Longitudinal-consistency screening excluded 14,465 children who did not maintain the same predefined correction-status and visual-acuity profile across all three visits. The final analytic cohort included 4152 children, including 391 in the ACG, 551 in the UCG, and 3210 in the UMG (Figure 1).

Baseline characteristics of initially eligible children included in and excluded from the final analytic cohort are presented in Supplementary Table S6. Compared with excluded children, those retained in the analytic cohort had less myopic SER, shorter AL, lower AL/CR, shallower ACD, and greater LT, with absolute SMDs ranging from 0.143 to 0.294. Differences in sex, age, K1, and K2 were small.

Baseline characteristics before matching are presented in Supplementary Table S1. A graded pattern was observed across the three groups: the UCG had the most myopic SER, the longest AL, and the highest AL/CR, followed by the ACG and then the UMG. The marked baseline imbalances in these characteristics supported the use of propensity score matching.

In the full cohort, the mean intervals were 287.2 ± 55.9 days from T0 to T1, 275.8 ± 83.7 days from T1 to T2, and 563.5 ± 74.3 days from T0 to T2. Group-specific and matched-cohort follow-up intervals are presented in Supplementary Table S2.

### 3.2. Covariate Balance After Propensity Score Matching

**Figure 2:**
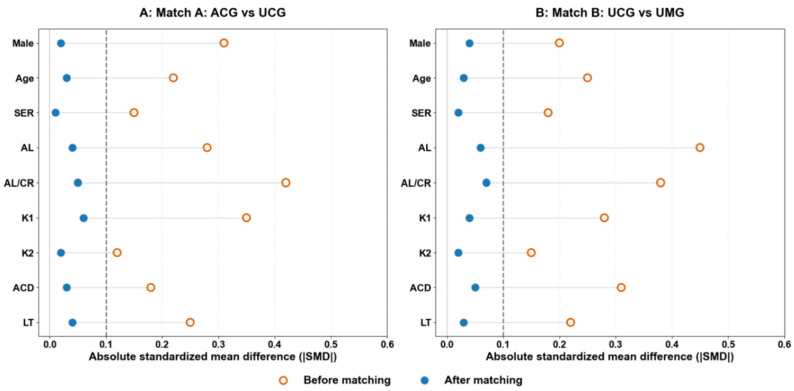
Covariate balance before and after propensity score matching in Match A (ACG vs. UCG) and Match B (UCG vs. UMG). SMD, standardized mean difference.

After 1:1 propensity score matching, Match A retained 313 of 391 ACG participants and 313 of 551 UCG participants, leaving 78 ACG and 238 UCG participants unmatched. Match B retained 387 of 551 UCG participants and 387 of 3210 UMG participants, leaving 164 UCG and 2823 UMG participants unmatched. Because matching was performed separately for the two comparisons, the matched UCG subsets were not identical. Propensity-score distributions indicated adequate common support before matching and improved overlap after matching (Supplementary Figure S1). All nine matching covariates achieved absolute SMDs < 0.10 after matching (Figure 2; Table 1).

### 3.3. Annualized Axial Length Growth After Propensity Score Matching

**Figure 3:**
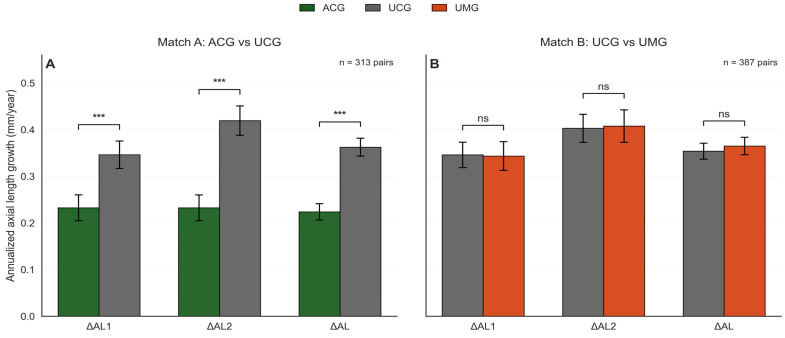
Annualized axial length growth in the propensity score-matched cohorts. (**A**) shows Match A, and (**B**) shows Match B. Bars represent means, and error bars represent 95% confidence intervals. *** *p* < 0.001; ns, not significant.

In Match A, ΔAL was significantly lower in the ACG than in the UCG (0.224 ± 0.158 vs. 0.362 ± 0.173 mm/year; mean difference, −0.139 mm/year; 95% CI, −0.165 to −0.112; *p* < 0.001). ΔAL1 and ΔAL2 were also lower in the ACG than in the UCG (Table 2; Figure 3A).

In Match B, no significant difference in ΔAL was observed between the UCG and UMG (0.354 ± 0.172 vs. 0.365 ± 0.188 mm/year; mean difference, −0.011 mm/year; 95% CI, −0.037 to 0.015; *p* = 0.412). Neither interval-specific outcome differed significantly between the groups (Table 2; Figure 3B).

### 3.4. Sensitivity Analyses

Across the examined caliper widths, all nine covariates remained adequately balanced, and the direction, magnitude, and statistical interpretation of the primary ΔAL estimates were unchanged in both matched comparisons (Supplementary Table S5).

**Figure 4:**
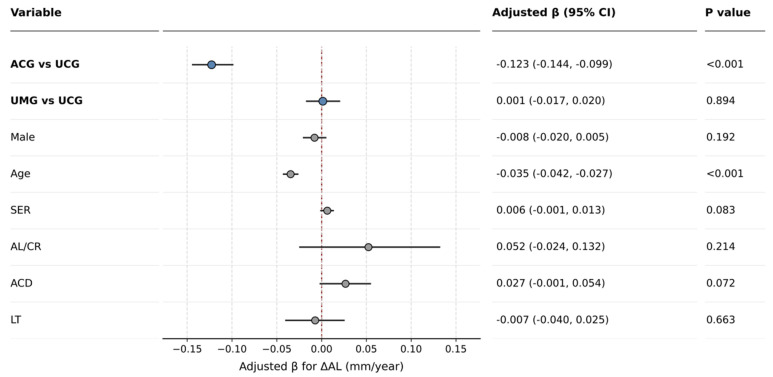
Full-cohort multivariable regression sensitivity analysis. Points represent adjusted regression coefficients and bootstrap 95% confidence intervals from the reduced multivariable model with ΔAL as the outcome. The UCG was the reference group; therefore, the group coefficients represent ACG versus UCG and UMG versus UCG. β, adjusted regression coefficient; CI, confidence interval.

In the full-cohort analysis, severe multicollinearity was identified among AL, AL/CR, K1, and K2. Because AL/CR incorporates both axial length and corneal curvature, including these closely related variables in the same model introduced overlapping information and made the individual regression coefficients difficult to interpret. The reduced model therefore retained AL/CR and excluded AL, K1, and K2. With the UCG as the reference group, the ACG remained associated with lower ΔAL (β = −0.123 mm/year; 95% CI, −0.144 to −0.099; *p* < 0.001), whereas the UMG did not differ significantly from the UCG (β = 0.001 mm/year; 95% CI, −0.017 to 0.020; *p* = 0.894). Older age was also associated with lower ΔAL (β = −0.035 mm/year per 1-year increase in age; 95% CI, −0.042 to −0.027; *p* < 0.001). Full regression results are presented in Figure 4 and Supplementary Table S3, with VIF diagnostics provided in Supplementary Table S4.

## 4. Discussion

This large real-world cohort study examined the association between stable habitual spectacle-corrected distance visual-acuity profiles and annualized axial length growth in school-based myopia screening. Children who maintained normal habitual spectacle-corrected distance visual acuity across three consecutive visits had lower annualized axial length growth than those with stable functional under-correction. No statistically significant difference was detected between the UCG and UMG. These findings suggest that achieved habitual corrected visual acuity provides information that is not captured by the binary record of spectacle wear alone.

This study differs from previous investigations of myopic under-correction in several important aspects. Prior studies have commonly defined under-correction according to the discrepancy between spectacle prescription and measured refractive status [7]. Such definitions are clinically meaningful but are difficult to apply in large-scale school-based screening when complete prescription records, lens condition, optical centration, and objective wearing behavior are unavailable. In the present study, the UCG was therefore an operational, performance-based category defined by consistently reduced habitual spectacle-corrected distance visual acuity rather than a prescription-defined diagnosis of refractive under-correction. Because non-cycloplegic autorefraction may be influenced by accommodation [8], annualized axial length growth was used as the primary outcome, and relatively stable ocular biometric parameters, including axial length, corneal curvature, and AL/CR, were included in the matching and regression analyses [9,10].

The baseline characteristics provide important context for interpreting the findings. Before matching, the UCG had the most myopic SER and the greatest AL and AL/CR, followed by the ACG and then the UMG. These imbalances indicate that spectacle-wearing status and achieved habitual visual acuity were associated with different baseline myopic profiles, supporting the need for propensity score matching. This observation is consistent with previous Chengdu screening data showing that the prevalence of full correction decreased with increasing myopia severity [1], but it should not be interpreted as evidence for the cause of reduced habitual corrected visual acuity in individual children. In addition, children retained in the final analytic cohort had less myopic SER, shorter AL, and lower AL/CR than those excluded, indicating that the final cohort represented a selected subgroup of initially eligible children.

The matched difference in ΔAL between the ACG and UCG was 0.139 mm/year, corresponding to a 38.4% lower annualized axial elongation rate in the ACG relative to the UCG. Over the mean T0–T2 interval of 563.5 days, this difference corresponds to approximately 0.21 mm less cumulative axial elongation, assuming a constant growth rate. This magnitude is not trivial for longitudinal axial-length monitoring. However, because no minimum clinically important difference was prespecified and this was an observational comparison, the estimate should not be interpreted as a causal myopia-control effect of achieving normal corrected visual acuity. In practice, persistently reduced habitual spectacle-corrected distance visual acuity in a child wearing spectacles may provide a reasonable basis for clinical reassessment, refraction, and evaluation of the current spectacles. The present study did not test whether using this measure improves referral decisions, prescription updating, or clinical outcomes.

One possible explanation is that better retinal image quality may reduce persistent blur and abnormal defocus signals involved in ocular growth regulation [11,12]. Conversely, reduced habitual visual quality in the UCG may reflect an outdated prescription, inconsistent spectacle wear, lens problems, refractive progression, or other causes, and may be associated with pathways implicated in axial elongation, including retinal dopamine signaling, choroidal responses, and scleral remodeling [13,14,15]. These mechanisms remain speculative and were not evaluated in the present study.

From a healthcare-delivery perspective, habitual spectacle-corrected distance visual acuity is inexpensive, scalable, and already collected in many school screening programs. Although repeated ocular biometry is informative for monitoring myopia progression, universal implementation at every screening point may be constrained by equipment, cost, personnel, and workflow capacity. Given that the long-term economic burden of myopia is related to disease progression and management strategies [16], and that current policy in China emphasizes strengthening myopia prevention and control at key stages in kindergartens and primary schools [17], habitual spectacle-corrected distance visual acuity may warrant prospective evaluation as an adjunct to follow-up prioritization. Further studies are needed to determine whether it provides incremental predictive value or improves screening, referral, and management outcomes.

This study has several strengths. First, it was based on a large real-world school-based screening database with three consecutive screening visits, allowing for correction status and visual-acuity profiles to be assessed longitudinally rather than at a single time point. Second, group membership was required to remain stable across all visits, reducing misclassification arising from transient changes in spectacle use, visual acuity, or measurement variability. Third, axial length growth was annualized according to actual examination intervals, reducing bias related to variation in follow-up timing. Fourth, the robustness of the findings was examined using propensity score matching, alternative caliper widths, interval-specific outcomes, and full-cohort multivariable regression.

Several limitations should be considered. First, restricting the analysis to children with stable correction-status and visual-acuity profiles produced a selected analytic cohort. Compared with excluded children, those retained had less myopic SER, shorter AL, and lower AL/CR. These differences limit generalizability to all initially eligible children. Second, propensity score matching balanced measured covariates only. Parental myopia, socioeconomic status, outdoor activity, near work, screen exposure, educational intensity, prescription age, lens condition, adherence, and access to eye care were unavailable and may have confounded the observed associations. Third, the UCG was an operational and heterogeneous category rather than a prescription-defined diagnosis. Reduced habitual corrected visual acuity could reflect an outdated prescription, inconsistent spectacle wear, damaged or poorly centered lenses, test variability, amblyopia, ocular surface disorders, or other ocular conditions. Fourth, non-cycloplegic SER may have been affected by accommodation, potentially influencing eligibility, matching, and regression adjustment, although axial length was used as the primary outcome. Fifth, right-eye-only analysis avoided inter-eye correlation but discarded fellow-eye information and may have reduced precision. Finally, the observational design does not support causal inference, and the absence of a statistically significant difference between the UCG and UMG should not be interpreted as evidence of equivalence.

## 5. Conclusions

Stable normal, habitual, spectacle-corrected distance visual acuity across three consecutive screening visits was associated with lower annualized axial length growth than stable functional under-correction in children with low-to-moderate myopia. In the primary matched comparison, the ACG had a 38.4% lower annualized axial elongation rate than the UCG, whereas no statistically significant difference was detected between the UCG and UMG. These findings suggest that achieved visual acuity under habitual spectacle-wearing conditions provides information beyond spectacle-wearing status alone. However, these observational results do not establish causality, predictive utility, equivalence between the UCG and UMG, or a myopia-control effect. Further prospective studies are needed to evaluate whether this routinely available measure can improve follow-up prioritization in school-based myopia screening.

## Figures and Tables

**Table 1:** Baseline characteristics of the propensity-score-matched cohorts.

Variable	Match A		|SMD|	Match B		|SMD|
	ACG	UCG		UCG	UMG	
Participants, n	313	313	—	387	387	—
Male, n (%)	182 (58.1%)	182 (58.1%)	0.000	190 (49.1%)	190 (49.1%)	0.000
Age (years)	8.930 ± 0.745	8.942 ± 0.794	0.016	8.924 ± 0.784	8.900 ± 0.750	0.032
SER (D)	−2.419 ± 1.010	−2.429 ± 0.981	0.011	−2.511 ± 0.937	−2.512 ± 1.071	0.001
AL (mm)	24.454 ± 0.760	24.428 ± 0.740	0.035	24.405 ± 0.740	24.376 ± 0.880	0.035
AL/CR	3.146 ± 0.091	3.147 ± 0.093	0.010	3.145 ± 0.089	3.144 ± 0.087	0.015
K1 (D)	42.816 ± 1.497	42.843 ± 1.483	0.019	42.893 ± 1.524	42.926 ± 1.634	0.021
K2 (D)	44.100 ± 1.636	44.190 ± 1.623	0.055	44.182 ± 1.646	44.240 ± 1.753	0.034
ACD (mm)	3.246 ± 0.238	3.236 ± 0.232	0.045	3.220 ± 0.235	3.228 ± 0.241	0.033
LT (mm)	3.432 ± 0.200	3.444 ± 0.197	0.059	3.441 ± 0.195	3.444 ± 0.212	0.012

ACG, adequately corrected group; UCG, functionally under-corrected group; UMG, uncorrected myopia group; SMD, standardized mean difference; SER, spherical equivalent refraction; AL, axial length; AL/CR, axial length-to-corneal radius ratio; K1/K2, flat and steep keratometry; ACD, anterior chamber depth; LT, lens thickness.

**Table 2:** Annualized axial length growth in the propensity score-matched cohorts.

**Match A. ACG vs. UCG**				
Outcome	ACG	UCG	Mean difference (95% CI)	*p* value
ΔAL1	0.232 ± 0.250	0.346 ± 0.265	−0.114 (−0.155 to −0.072)	<0.001
ΔAL2	0.232 ± 0.249	0.419 ± 0.283	−0.187 (−0.230 to −0.144)	<0.001
ΔAL	0.224 ± 0.158	0.362 ± 0.173	−0.139 (−0.165 to −0.112)	<0.001
**Match B. UCG vs. UMG**				
Outcome	UCG	UMG	Mean difference (95% CI)	*p* value
ΔAL1	0.346 ± 0.272	0.343 ± 0.307	0.002 (−0.039 to 0.043)	0.908
ΔAL2	0.403 ± 0.301	0.408 ± 0.349	−0.005 (−0.053 to 0.043)	0.844
ΔAL	0.354 ± 0.172	0.365 ± 0.188	−0.011 (−0.037 to 0.015)	0.412

All outcome values and mean differences are expressed in mm/year. ΔAL1, annualized axial length growth from baseline to the first follow-up; ΔAL2, annualized axial length growth from the first follow-up to the second follow-up; ΔAL, overall annualized axial length growth from baseline to the second follow-up; CI, confidence interval.

## Data Availability

The datasets analyzed in this study are not publicly available because they contain individual-level school-based screening data from minors and are subject to ethical and privacy restrictions. De-identified data may be made available from the corresponding author upon reasonable request and with approval from the relevant ethics committee or data governance authority.

## Supplementary Materials

The following supporting information can be downloaded at https://www.mdpi.com/article/10.3390/healthcare14142076/s1: Table S1: Baseline characteristics before propensity-score matching; Table S2: Follow-up intervals in the full analytic cohort and propensity-score-matched cohorts; Figure S1: Propensity-score distributions before and after matching; Table S3: Full-cohort multivariable regression sensitivity analysis for annualized axial length growth; Table S4: Variance inflation factor diagnostics for the full multivariable model; Table S5: Sensitivity of propensity-score-matched estimates to alternative caliper widths; Table S6: Baseline characteristics of initially eligible children included in and excluded from the final analytic cohort.

## Abbreviations

The following abbreviations are used in this manuscript:

ACG	adequately corrected group
UCG	functionally under-corrected group
UMG	uncorrected myopia group
SVS	single-vision spectacles
SER	spherical equivalent refraction
AL	axial length
AL/CR	axial length-to-corneal radius ratio
CDVA	corrected distance visual acuity
UCVA	uncorrected distance visual acuity
K1/K2	flat/steep keratometry
ACD	anterior chamber depth
LT	lens thickness
PSM	propensity score matching
SMD	standardized mean difference
CI	confidence interval
VIF	variance inflation factor
ΔAL1	annualized axial length growth from baseline to the first follow-up
ΔAL2	annualized axial length growth from the first follow-up to the second follow-up
ΔAL	overall annualized axial length growth from baseline to the second follow-up
