# Supplementary material for: Habitual Spectacle-Corrected Distance Visual Acuity and Axial Elongation in School-Based Myopia Screening: A Retrospective Cohort Study
Source: Healthcare (Basel). 2026 Jul 10;14(14):2076. doi: 10.3390/healthcare14142076 (PMC13412033; doi:10.3390/healthcare14142076)
Supplement: Supplementary file 1 [file healthcare-14-02076-s001.zip › healthcare-4383718-supplementary.pdf]

Supplementary Materials

Supplementary Table S1. Baseline characteristics before propensity-score matching

Variable	Match A: ACG vs UCG				Match B: UCG vs UMG			
	ACG (n = 391)	UCG (n = 551)	P value	Absolute SMD	UCG (n = 551)	UMG (n = 3,210)	P value	Absolute SMD
Male sex, n (%)	228 (58.3%)	276 (50.1%)	0.015	0.166	276 (50.1%)	1,630 (50.8%)	0.801	0.014
Age, years	8.913 ± 0.756	8.961 ± 0.770	0.344	0.063	8.961 ± 0.770	8.714 ± 0.823	<0.001	0.309
CDVA, logMAR	−0.003 ± 0.018	0.205 ± 0.149	<0.001	1.959	—	—	—	—
SER, D	−2.144 ± 1.078	−2.963 ± 1.140	<0.001	0.738	−2.963 ± 1.140	−1.363 ± 0.799	<0.001	1.625
AL, mm	24.328 ± 0.804	24.583 ± 0.775	<0.001	0.322	24.583 ± 0.775	23.784 ± 0.808	<0.001	1.009
AL/CR	3.128 ± 0.103	3.173 ± 0.095	<0.001	0.458	3.173 ± 0.095	3.059 ± 0.088	<0.001	1.246
K1, D	42.752 ± 1.529	42.938 ± 1.481	0.063	0.123	42.938 ± 1.481	42.871 ± 1.499	0.330	0.045
K2, D	44.115 ± 1.627	44.280 ± 1.597	0.122	0.102	44.280 ± 1.597	44.042 ± 1.625	0.001	0.147
ACD, mm	3.216 ± 0.244	3.238 ± 0.233	0.156	0.094	3.238 ± 0.233	3.127 ± 0.264	<0.001	0.447
LT, mm	3.440 ± 0.197	3.431 ± 0.187	0.492	0.046	3.431 ± 0.187	3.520 ± 0.230	<0.001	0.423

Values are mean ± standard deviation unless otherwise indicated. CDVA was not included in the propensity-score model because it defined correction-status groups. ACG, adequately corrected group; UCG, functionally under-corrected group; UMG, uncorrected myopia group; CDVA, corrected distance visual acuity; logMAR, logarithm of the minimum angle of resolution; SMD, standardized mean difference; SER, spherical equivalent refraction; AL, axial length; AL/CR, axial length-to-corneal radius ratio; K1 and K2, flat and steep keratometry, respectively; ACD, anterior chamber depth; LT, lens thickness.

Supplementary Table S2. Follow-up intervals in the full analytic cohort and propensity-score-matched cohorts

Panel A. Full analytic cohort

Follow-up interval, days	ACG (n = 391)	UCG (n = 551)	UMG (n = 3,210)	Overall (n = 4,152)
T0–T1	293.8 ± 33.5	268.4 ± 19.5	289.6 ± 61.4	287.2 ± 55.9
T1–T2	269.8 ± 52.7	251.2 ± 56.6	280.8 ± 89.7	275.8 ± 83.7
T0–T2	564.2 ± 40.0	520.2 ± 42.4	570.8 ± 79.1	563.5 ± 74.3

Panel B. Match A: ACG vs UCG

Follow-up interval, days	ACG (n = 313)	UCG (n = 313)	Paired mean difference (95% CI), days
T0–T1	287.5 ± 27.5	271.9 ± 24.9	15.6 (13.1 to 18.1)
T1–T2	264.2 ± 51.5	251.7 ± 59.5	12.5 (6.9 to 18.1)
T0–T2	552.3 ± 41.7	524.2 ± 49.5	28.1 (23.8 to 32.4)

Panel C. Match B: UCG vs UMG

Follow-up interval, days	UCG (n = 387)	UMG (n = 387)	Paired mean difference (95% CI), days
T0–T1	266.0 ± 18.6	270.2 ± 33.0	−4.2 (−6.9 to −1.5)
T1–T2	248.0 ± 47.5	250.3 ± 65.0	−2.3 (−7.6 to 3.0)
T0–T2	514.6 ± 33.3	521.1 ± 42.4	−6.5 (−9.9 to −3.1)

Values are mean ± standard deviation. Paired mean differences were calculated as ACG minus UCG in Match A and UCG minus UMG in Match B. T0, baseline; T1, first follow-up; T2, second follow-up; CI, confidence interval.

Supplementary Figure S1. Propensity-score distributions before and after matching

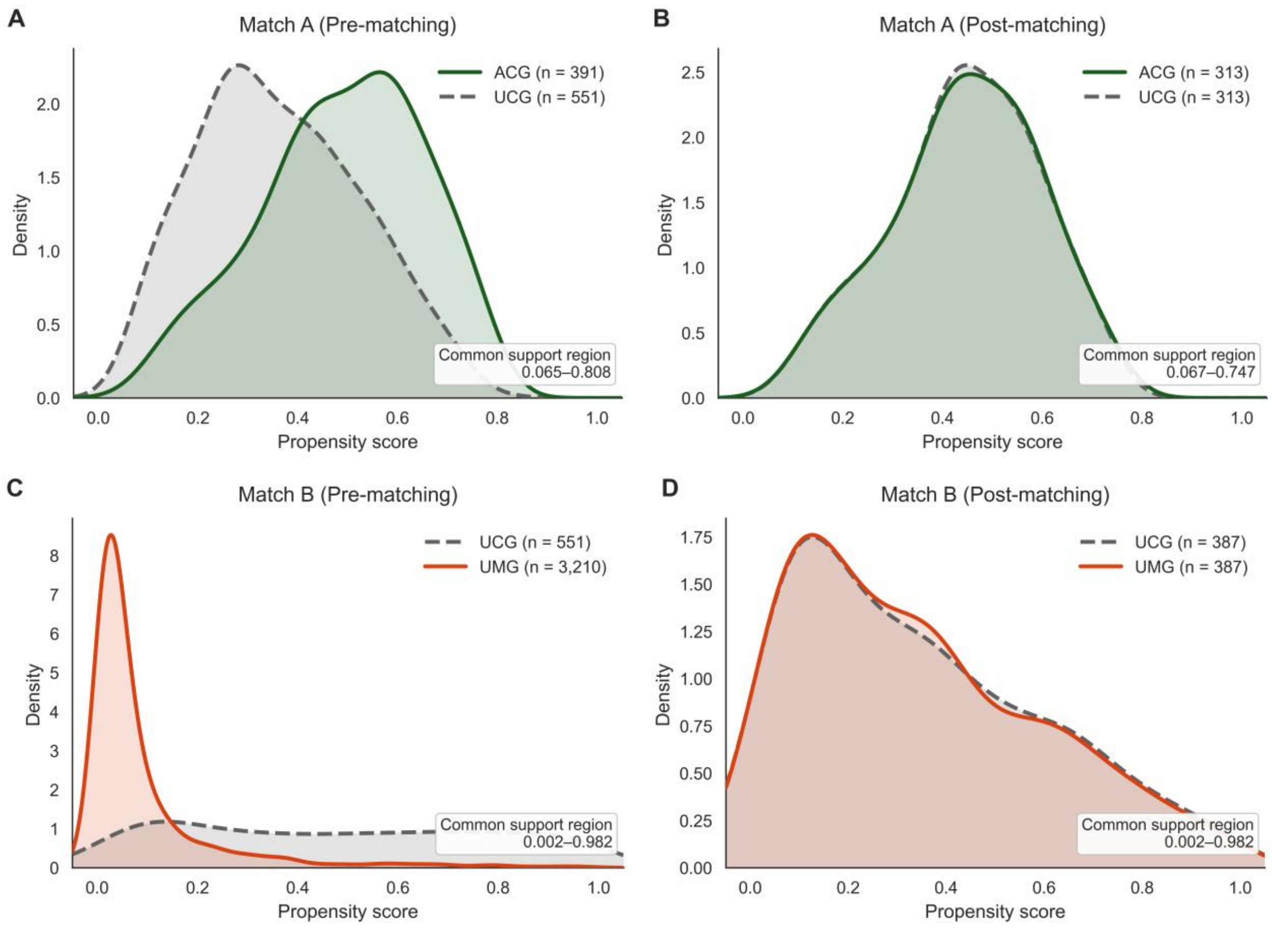

Panels A and B show the propensity-score distributions before and after 1:1 matching for Match A (ACG vs UCG), respectively. Panels C and D show the corresponding distributions for Match B (UCG vs UMG). The empirical common-support region was defined as the overlap between the observed propensity-score ranges of the two comparison groups. Substantial overlap was observed after matching in both comparisons.

Supplementary Table S3. Full-cohort multivariable regression sensitivity analysis for annualized axial length growth

Variable	Adjusted β	SE	95% CI	P value
**Correction status (reference = UCG)**				
ACG vs UCG	−0.123	0.012	−0.144 to −0.099	<0.001
UMG vs UCG	0.001	0.010	−0.017 to 0.020	0.894
**Baseline covariates**				
Male sex	−0.008	0.006	−0.020 to 0.005	0.192
Age	−0.035	0.004	−0.042 to −0.027	<0.001
SER	0.006	0.004	−0.001 to 0.013	0.083
AL/CR	0.052	0.040	−0.024 to 0.132	0.214
ACD	0.027	0.015	−0.001 to 0.054	0.072
LT	−0.007	0.016	−0.040 to 0.025	0.663

The reduced model included correction status, sex, age, SER, AL/CR, ACD, and LT; UCG served as the reference group. AL, K1, and K2 were excluded because of severe collinearity in the full model. β, adjusted regression coefficient; SE, standard error.

Supplementary Table S4. Variance inflation factor diagnostics for the full multivariable model

Variable	VIF	Interpretation
AL	573.918	Severe collinearity
AL/CR	465.237	Severe collinearity
K2	152.085	Severe collinearity
K1	152.063	Severe collinearity
UMG vs UCG	2.250	Acceptable
ACD	1.895	Acceptable
SER	1.845	Acceptable
LT	1.643	Acceptable
ACG vs UCG	1.629	Acceptable
Male sex	1.189	Acceptable
Age	1.138	Acceptable

VIF, variance inflation factor. VIF values <5 were considered acceptable, values from 5 to <10 moderate, and values ≥10 indicative of severe collinearity.

Supplementary Table S5. Sensitivity of propensity-score-matched estimates to alternative caliper widths

Caliper width, SD of logit PS	Absolute caliper width on logit-PS scale	Matched pairs, n	Maximum post-match absolute SMD	Covariates with absolute SMD < 0.10, n/9	ACG ΔAL, mm/year	UCG ΔAL, mm/year	Paired mean difference (95% CI), mm/year	P value
**Panel A. Match A: ACG vs UCG**								
0.10	0.082655	306	0.061	9/9	0.224 ± 0.160	0.365 ± 0.173	−0.141 (−0.168 to −0.114)	<0.001
0.15	0.123983	310	0.072	9/9	0.225 ± 0.158	0.363 ± 0.173	−0.139 (−0.165 to −0.112)	<0.001
0.20†	0.165310	313	0.059	9/9	0.224 ± 0.158	0.362 ± 0.173	−0.139 (−0.165 to −0.112)	<0.001
0.25	0.206638	317	0.063	9/9	0.223 ± 0.159	0.364 ± 0.173	−0.140 (−0.166 to −0.114)	<0.001

Caliper width, SD of logit PS	Absolute caliper width on logit-PS scale	Matched pairs, n	Maximum post-match absolute SMD	Covariates with absolute SMD < 0.10, n/9	UCG ΔAL, mm/year	UMG ΔAL, mm/year	Paired mean difference (95% CI), mm/year	P value
**Panel B. Match B: UCG vs UMG**								
0.10	0.179042	379	0.038	9/9	0.358 ± 0.168	0.366 ± 0.188	−0.008 (−0.035 to 0.018)	0.549
0.15	0.268562	384	0.034	9/9	0.354 ± 0.172	0.364 ± 0.188	−0.010 (−0.037 to 0.017)	0.462
0.20†	0.358083	387	0.035	9/9	0.354 ± 0.172	0.365 ± 0.188	−0.011 (−0.037 to 0.015)	0.412
0.25	0.447604	391	0.039	9/9	0.356 ± 0.172	0.364 ± 0.188	−0.008 (−0.034 to 0.019)	0.572

Values for ΔAL are mean ± standard deviation. All analyses used the same propensity-score model, exact matching on sex, and greedy 1:1 nearest-neighbor matching without replacement; only the caliper width was varied. Caliper widths are expressed as multiples of the standard deviation of the logit of the propensity score calculated in each pre-matching comparison sample. The primary analysis used a caliper width of 0.20 and is marked with a dagger (†). Mean differences were calculated as ACG minus UCG in Match A and UCG minus UMG in Match B. Maximum post-match absolute SMD denotes the largest absolute standardized mean difference among the nine matching covariates. SD, standard deviation; PS, propensity score; ΔAL, annualized axial length growth from baseline to the second follow-up.

Supplementary Table S6. Baseline characteristics of initially eligible children included in and excluded from the final analytic cohort.

Variable	Included cohort	Excluded cohort	P value	|SMD|
Participants, n	4,152	14,465	–	–
Male sex, n (%)	2134 (51.4%)	7248 (50.1%)	0.143	0.026
Age, years	8.766 ± 0.815	8.776 ± 0.819	0.476	0.013
SER, D	−1.649 ± 1.044	−1.914 ± 1.071	<0.001	0.251
AL, mm	23.941 ± 0.856	24.094 ± 0.836	<0.001	0.181
AL/CR	3.081 ± 0.099	3.111 ± 0.103	<0.001	0.294
K1, D	42.869 ± 1.500	42.857 ± 1.537	0.689	0.007
K2, D	44.081 ± 1.623	44.062 ± 1.647	0.548	0.011
ACD, mm	3.150 ± 0.262	3.193 ± 0.253	<0.001	0.165
LT, mm	3.501 ± 0.224	3.469 ± 0.213	<0.001	0.143

Values are mean ± standard deviation unless otherwise indicated. P values were calculated using Welch's independent-samples t tests for continuous variables and Pearson's chi-square test for sex. Absolute standardized mean differences were calculated to quantify between-group differences.
